# Case of Glanders in the Human Subject

**Published:** 1868-12

**Authors:** W. O. Baldwin

**Affiliations:** Montgomery, Alabama


### CASE OF GLANDERS IN THE HUMAN SUBJECT.

By W. O. BALDWIN, M.D., Montgomery, Alabama.

[Read before the Montgomery Medical and Surgical Society.]

On the evening of the 11th of May last, I received a message from Drs. Tompkins, Townsend, and Roberts, requesting me to visit, in consultation with them, Mr. Eli Townsend, a highly respectable citizen, living at China Grove, Pike County—distant about 38 miles, whom they supposed to have glanders. I went, and on reaching the place late in the afternoon of the 12th, I met the physicians named above, from whom, together with the family, I received the following history of the case, of which I made a record before leaving the house, and in the presence of the physicians:—

Mr. Townsend is a planter, in easy circumstances, now about 71 years of age; of large and once powerful frame; of active and industrious habits; has always enjoyed excellent health, and up to the commencement of this attack was in remarkable preservation for one of his years. He has had on his premises a very fine saddle-horse, affected with glanders since Christmas last. This horse he has kept in a close stable on his premises, but apart from the balance of his stock, and has persisted in tending him himself, in spite of the remonstrances of his family. About the 27th of April he received a small abrasion on the dorsum of the left hand. He continued, however, to nurse his horse, and among other duties which he performed for him was that of daily syringing his nostrils with vinegar and water. The injected fluid, on its return, was of course mingled with the secretions from the nose of the animal, which were said to be very abundant, and it is quite likely that more or less of these offensive matters came in contact with the abraded surface on the hand during some of these operations with the syringe. He seemed to have some apprehension in exposing his hands in this way after the skin had become broken, and though he continued to use the syringe daily, he never failed to go immediately to the house, where, after giving his hands a thorough washing with soap and water, he would apply a small bit of paper saturated with spirits of turpentine over the abraded surface, as a preventive of bad consequences.

On the night of the 4th ult., about seven days after the abrasure on the hand, he felt a very slight uneasiness about the spot. On the next day (5th) he visited a relation in the neighborhood, where he spent the day, and whilst there observed that the uneasiness in wound on hand had increased, and noticed red streaks running up the arm, with some pain in axilla of corresponding side. Complained, also, of great oppression and distention about stomach, and when the dinner hour arrived could not eat. Had no absolute pain in stomach, but, from the general distress in that region, said he thought he must have colic. Returned home in afternoon, and had rigors and flushes of heat, succeeded by fever. At night took a large dose of calomel.

On the 6th calomel operated several times, but distress about the stomach still continued, accompanied with nausea. Hand and whole arm considerably swollen; some spots on skin hard to the touch, and as "red as red flannel" on both arm and forearm. Had alternate rigors and fever, with great restlessness. Took several small doses of calomel.

On the 7th all the symptoms increased in violence. Took a dose of Cooke's pills. On the 8th, whilst applying a poultice to the affected arm, the nurse noticed that one of the hardest tumors on the forearm was discharging a thin serous fluid, and that underneath the cuticle where it had been broken the tissues presented a very dark, blackened appearance.

Up to this time he had not been visited by a physician. On the 9th he was seen by Dr. Tompkins, a highly intelligent physician of the neighborhood, who immediately diagnosed his condition as that rare disease in the human subject, known as glanders, which is sometimes communicated to man by the horse, ass, or mule. Pulse full and regular, and about 90 per minute; skin hot and dry; tongue heavily loaded, and moist; general feeling of uneasiness, distress, and restlessness, with pains and soreness in extremities; thirst and nausea, but no vomiting or retching; rigors occasionally, and a desire to get out of bed and sit by the fire, which he sometimes did. Arm and forearm much swollen, with a general erysipelatous look; pustules, tubercles, and bullæ with hardened bases interspersed on different portions of limb, of varying sizes and shapes and in various stages of development, one of which had broken and was discharging a thin sanious fluid, and revealing under the cuticle a blackish, sphacelated mass. Original wound where the skin had been abraded on back of hand, and which had been the point of inoculation, and now about the size of a twenty-five cent piece, somewhat sunken, of a dark leaden color, and emitting a thin sanious fluid. Prescribed chlorate potassa internally and lead lotion to arm.

Drs. Roberts and Townsend saw the case in consultation on the 10th. Their description of his condition on that day did not differ from the one given by Dr. Tompkins, of his symptoms on the 9th, except in the general progress towards virulence and malignancy.

On the 11th and 12th there seemed great tendency towards congestion in the mornings, marked by coldness of extremities, a leaden pallor of surface, and feebleness of pulse. This condition was followed in about four hours with febrile reaction, but without increase of frequency of pulse, which has been tolerably uniform at 98 per minute; has been freely purged. Commenced taking calomel and opium on the 10th, which was continued at intervals until 12 o'clock M. on the 12th. Nitrate of silver was applied to wound, over and around tumors and gangrenous spots, and heavy caustic lines drawn around arm in front of axilla.

When seen by me at 6 o'clock P.M. on the 12th ult., pulse 71, large, bounding, and intermittent; skin moderately hot, and occasional free perspirations; tongue dry, and considerably coated; great hebetude of intellect (this, I afterwards thought, was to some extent due to a pretty full dose of morphine taken in the early part of day, as when I left him next morning his mind was clear). Has hiccoughs, some nausea, thirst, loathing of food, and extreme jactitation. Left arm somewhat swollen and red. The original wound at point of inoculation about an inch in diameter, circular, in a sloughy condition, and discharging a thin, dirty-looking serum. On other portions of the limb there were spots of different sizes and stages of development, from some of which the cuticle had been denuded, leaving a dark, almost black appearance, from which a dark-reddish fluid exuded. One of these gangrenous tubercles was situated midway and on back of left forearm, about three inches in length and one and a half inches in width, from which the cuticle had slipped off as from a burn, leaving a dark gangrenous mass underneath, which was discharging a thin, dark-red sanies. On the right arm, over the olecranon process, there was observed on yesterday a small, hard, red spot, which is now about three or four inches in diameter, and circular, the cuticle over which presents the appearance of a blistered surface, and the skin underneath (which can be seen where the cuticle has been torn in a small place) of a dark, livid color. These tumors or tubercles, when they are first observed, are only red spots upon the skin, which is somewhat elevated, with a hardened base. Within 36 or 48 hours they assume the appearance of regular bullæ, the cuticle looking as if it had been scalded, beneath which a thin fluid collects into a vesicle embracing the extent of the hardened base, easily broken or destroyed, and revealing the sphacelated tissues underneath, varying in appearance from a livid color to that characterizing complete gangrene, according to their duration—the latter generally occurring within 48 hours after first appearance. One of these hardened tumors, which was pointed out to me, located near the elbow of the left arm, seemed to have resulted in the formation of a small abscess, which had discharged 36 hours after the discoloration and elevation in the skin had been first observed. The discharge from this point was described as "considerable," and consisting of a bloody fluid mixed with quantities of matter having somewhat the characteristics of genuine pus. The tissues around this particular spot were the only ones which did not present either a sphacelus, or a decided tendency towards gangrene, in all places where the cuticle had been broken.

On different portions of the arm and forearm there are scattered three or four pustules, resembling fully-matured small-pox vesicles, but larger and more pointed. One of these pustules was also found on the breast, and another on one of the legs of the patient. The prescription, which was the result of one consultation, was the avoidance of all exhausting remedies and the free use of sustaining measures, such as animal broths, milk, rich wines, brandy, etc., together with the internal use of chlorate of potassa with hydrochloric acid and some preparation of iron. Prognosis unfavorable.

I left a request that the physicians in attendance would furnish me notes on the subsequent progress of the case, which was very promptly complied with by Dr. Roberts. These being full, embrace a considerable space, and I do not deem it important to read them here, further than to give a general synopsis of them.

The march of the disease was characterized by pretty much the same symptoms enumerated in the above report, giving at no time any indication of amelioration, but, on the contrary, marked by a progressive increase in violence. Patient became quite delirious on the night of the 13th, which continued in a greater or less degree, with low muttering and coma towards the close. Involuntary discharges of urine came on. Hiccough, with dryness and redness of the tongue and mouth, and excessive thirst, were present throughout. Old abscesses and gangrenous spots enlarged and sloughed. Four abscesses formed, and pustules scattered over the limbs, body, and face, varying in size from that of a pea to a hazlenut. Eyes became swollen, with a livid discoloration surrounding them, with an unusual trembling of lower jaw. Jactitation, which had been excessive throughout the attack, continued, with subsultus tendinum, until the patient expired, on the morning of the 19th, about fifteen days after the beginning of the attack. No autopsy was made. The limb, which was much swollen at first, became greatly reduced in size.

The transmissibility of disease from the equine tribes of animals to the human subject, though once questioned, has been demonstrated beyond the possibility of a doubt. I have not been able to find any records touching the subject of glanders in man, going farther back than the present century, and it may, therefore, be said to be comparatively a new disease upon our calendar; and it is more in consequence of the novelty of the disease itself, than from any special points of interest possessed by this particular case, that I have been induced to read you the above notes. This is the first case of glanders in the human subject I ever saw, and I have never before heard of a case in this State. The first case on record, which I have seen noticed, was that reported by Muscroft, of Edinburgh, in 1821, and the second reported by Dr. Rimu, of Germany, in 1832. To Dr. Elliotson, however, is due the credit of having done more, in the investigation of the subject, than perhaps any other man.

In order to indicate something of its nature, as well as to define its origin, the latter gentleman has applied to the disease in the human subject the term *equinia*, which has been adopted and qualified by other authors, so as to include another affection also communicated from the horse to the human subject. This is characterized by a pustular eruption, and occasionally communicated to coachmen and stable-boys who tend and dress the heels of horses affected by the disease peculiar to that animal, and known as *grease*. To the latter the term *equinia mites* has been applied; whilst to the one emanating from the glandered horse the term *equinia glandulosa* has been assigned.

According to Dr. Elliotson, equinia glandulosa may appear in the human subject in different forms:—1. In that of simple acute glanders; the disease attacking the nasal cavities and adjoining parts. 2. In that of acute *farcy glanders*, appearing in various parts, in the form of small tumors, which suppurate and give rise to foul ulcers. 3. Where varieties may exist separately, or they may be both produced at the same time, or the one may precede the other. 4. Each of these varieties may also occur in a chronic form; they may also exist separately, or be conjoined. Besides these divisions of the disease, other subdivisions have been made. Thus, Rayer distinguishes in the acute form an ecchymotic, a gangrenous, and a pustular variety—all agreeing, however, that these different varieties constitute one and the same disease, and are produced by the same specific cause, and may all occur in the same case.

In the case here detailed it will be observed that a set of symptoms, which is, perhaps, the most characteristic of all those which usually attend glanders of the human subject, was entirely absent. I allude, of course, to those affecting the pituitary membrane. In many particulars there was a close resemblance between the symptoms in this case and those which attend severe and aggravated cases of haimatoxic poisoning from dissecting wounds; and, in the absence of the specific contagion to which it was known the patient had been exposed, it might very properly have been classed under that head.

In speaking of the diagnosis, Dr. Gross says:—"From the effects of a dissection wound, it may be readily distinguished by the peculiar discharges from the nose and by the character of the cutaneous eruption. The history of the case, too, will furnish important diagnostic data, and should, therefore, always receive due consideration. The fact that the patient has nursed or examined a glandered horse or person will, generally of itself, afford strong presumptive proof of the true character of the attack." The nasal discharge, as is said to have been the case in more than two-thirds of all the cases on record, was in this instance entirely wanting, whilst the character of the cutaneous eruptions did not differ materially from such as are often opened in dissecting wounds; so that the only means of diagnosis left us in some cases, and, particularly, as between some cases of dissection wounds and farcy glanders, is in relation with our knowledge of the cause and circumstances of the attack. The original wound at the point of inoculation, with its red streaks running to the axilla, the general erysipelatous appearance of the limb, the vesicles or bullæ appearing over the red, hardened tumors on different portions of the limb and body, with thin, sub-cuticular, gangrenous sloughs, together with the pustules and abscesses, first appearing on the diseased limb and finally extending to every portion of the body, create an assemblage of spmptoms so closely resembling each other in the two diseases, that I must confess I should hesitate to decide from this data alone upon the diagnosis. The constitutional symptoms also are scarcely less similar than those of the surface; and the general progress of the two diseases is marked by pretty much the same phenomena—except that in one the disease is generally fatal, whilst in the other the reverse is the rule. So that at last our only positive means of diagnosis, added to the symptoms common to both, is our knowledge as to whether or not the patient has been exposed to the contagion in the one instance, or the producing cause in the other.

I have not intended to read you an essay on glanders, but simply to give you the facts, circumstances, and symptoms attending what seemed to me to be a very rare and interesting case of disease. I have done this with the more pleasure because I know that some members of the profession, and, perhaps, some members of this Society, do not believe in the existence of such a disease as glanders in the human subject. I feel entirely satisfied that this was the specific disease known as glanders, reproduced in the human subject, through inoculation from the horse. One of the features of the disease, which is the most characteristic where it does occur, was not present, it is true

(I allude, of course, to the nasal symptoms), but yet it is shown that this has been the case with a large majority of the cases which have been recorded, and that its existence is by no means necessary in order to constitute a case of glanders. Some authors have contended that glanders and farcy are two separate and distinct diseases, whilst the weight of evidence is strongly in favor of their identity. This latter view gains confirmation from this case, from the fact that it was communicated from an animal in which the prominent features of the disease were those claimed for glanders; whilst it reproduced in the human subject inoculated from it only those phenomena common to farcy.

Note.—This paper, contributed by Dr. W. O. Baldwin, the present President of the American Medical Association will, it is hoped, suggest inquiry and stimulate investigation, in regard to this interesting subject. All information communicated will be published with pleasure.—Ed.—*Richmond and Louisville Medical Journal.*

